# Donanemab Appropriate Use Recommendations

**DOI:** 10.1002/alz70859_106086

**Published:** 2025-12-25

**Authors:** Gil D. Rabinovici

**Affiliations:** ^1^ UCSF Alzheimer's Disease Research Center, San Francisco, CA, USA; Memory and Aging Center, Weill Institute for Neurosciences, University of California San Francisco, San Francisco, CA USA

## Abstract

**Background:**

Donanemab (Kisunla ®), an IgG1 monoclonal antibody targeting the pyroglutamate‐modified form of amyloid‐β, is approved in the United States and other countries for treatment of early‐stage Alzheimer’s disease (AD). The Appropriate Use Recommendations (AUR) were developed to guide the implementation of donanemab in real‐world practice, prioritizing safety considerations and opportunity for effectiveness.

**Methods:**

The AUR were developed by the AD and Related Disorders Therapeutic Workgroup, integrating available data and expert opinion. Workgroup members reviewed all publicly available data regarding the drug’s efficacy and safety profile, safety data pertaining to other Aβ‐targeting antibodies, as well as expert opinion. Group deliberations took place between October 2023 and November 2024 via video conferences and written exchanges, with reiterative refinement until consensus was achieved.

**Results:**

Appropriate candidates for donanemab include persons with mild cognitive impairment or mild dementia due to AD (Clinical Stages 3‐4, MMSE 20‐30) who show biomarker support for AD by PET or CSF. Tau PET is not required for eligibility, though, if available, can be considered to individualize estimates of likely clinical response. Apolipoprotein E genotyping is required prior to treatment to inform an individual’s risk of developing Amyloid Related Imaging Abnormalities (ARIA). Pre‐treatment MRI should be obtained ≤ 12 months prior to treatment, and patients with findings of significant cerebral amyloid angiopathy or a major vascular contribution to cognitive impairment should be excluded. The decision to initiate therapy should be grounded in a shared decision‐making process that emphasizes the patient’s values and goals of care. Donanemab is administered as a monthly intravenous infusion. MRI scans to evaluate for ARIA should be performed prior to the 2^nd^, 3^rd^, 4^th^ and 7^th^ infusions, and at any time ARIA is suspected clinically. Clinicians may consider discontinuing treatment if amyloid clearance is demonstrated by amyloid PET 12‐18 months after initiating treatment.

**Conclusions:**

The AUR adapt the donanemab clinical trial criteria and protocol into clinical practice, in order to maximize the safe and efficacious translation of this novel therapy into patient care. The AUR are expected to evolve as more real‐world data accumulate from ALZNET and other patient registries in the U.S. and globally.

